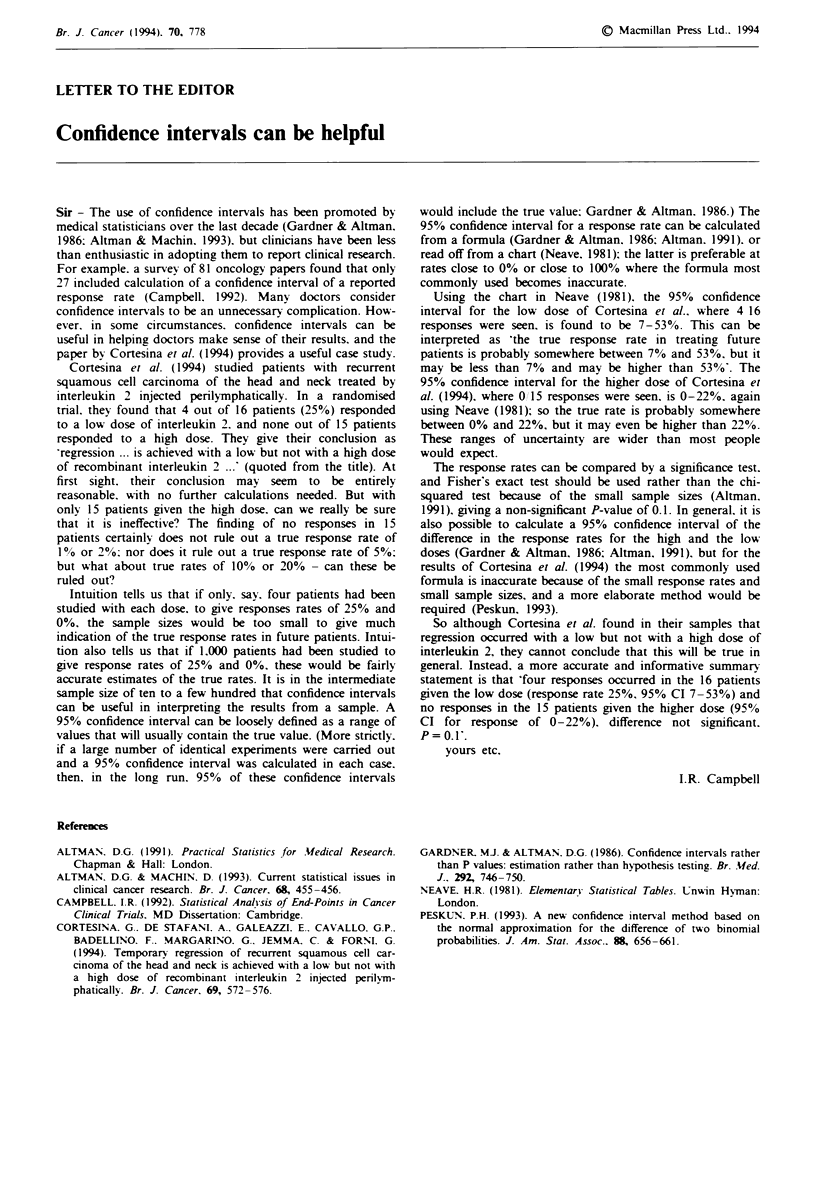# Confidence intervals can be helpful.

**DOI:** 10.1038/bjc.1994.396

**Published:** 1994-10

**Authors:** I. R. Campbell


Br. J. Cancer (1994), 70, 778 © Macmillan Press Ltd., 1994

### LETTER TO THE EDITOR

### Confidence intervals can be helpful

**Sir** – The use of confidence intervals has been promoted by medical statisticians over the last decade (Gardner & Altman, 1986; Altman & Machin, 1993), but clinicians have been less than enthusiastic in adopting them to report clinical research. For example, a survey of 81 oncology papers found that only 27 included calculation of a confidence interval of a reported response rate (Campbell, 1992). Many doctors consider confidence intervals to be an unnecessary complication. However, in some circumstances, confidence intervals can be useful in helping doctors make sense of their results, and the paper by Cortesina *et al.* (1994) provides a useful case study.

Cortesina *et al.* (1994) studied patients with recurrent squamous cell carcinoma of the head and neck treated by interleukin 2 injected perilymphatically. In a randomised trial, they found that 4 out of 16 patients (25%) responded to a low dose of interleukin 2, and none out of 15 patients responded to a high dose. They give their conclusion as 'regression ... is achieved with a low but not with a high dose of recombinant interleukin 2 ...' (quoted from the title). At first sight, their conclusion may seem to be entirely reasonable, with no further calculations needed. But with only 15 patients given the high dose, can we really be sure that it is ineffective? The finding of no responses in 15 patients certainly does not rule out a true response rate of 1% or 2%; nor does it rule out a true response rate of 5%; but what about true rates of 10% or 20% – can these be ruled out?

Intuition tells us that if only, say, four patients had been studied with each dose, to give responses rates of 25% and 0%, the sample sizes would be too small to give much indication of the true response rates in future patients. Intuition also tells us that if 1,000 patients had been studied to give response rates of 25% and 0%, these would be fairly accurate estimates of the true rates. It is in the intermediate sample size of ten to a few hundred that confidence intervals can be useful in interpreting the results from a sample. A 95% confidence interval can be loosely defined as a range of values that will usually contain the true value. (More strictly, if a large number of identical experiments were carried out and a 95% confidence interval was calculated in each case, then, in the long run, 95% of these confidence intervals would include the true value; Gardner & Altman, 1986.) The 95% confidence interval for a response rate can be calculated from a formula (Gardner & Altman, 1986; Altman, 1991), or read off from a chart (Neave, 1981); the latter is preferable at rates close to 0% or close to 100% where the formula most commonly used becomes inaccurate.

Using the chart in Neave (1981), the 95% confidence interval for the low dose of Cortesina *et al.*, where 4/16 responses were seen, is found to be 7–53%. This can be interpreted as 'the true response rate in treating future patients is probably somewhere between 7% and 53%, but it may be less than 7% and may be higher than 53%'. The 95% confidence interval for the higher dose of Cortesina *et al.* (1994), where 0/15 responses were seen, is 0–22%, again using Neave (1981); so the true rate is probably somewhere between 0% and 22%, but it may even be higher than 22%. These ranges of uncertainty are wider than most people would expect.

The response rates can be compared by a significance test, and Fisher's exact test should be used rather than the chi-squared test because of the small sample sizes (Altman, 1991), giving a non-significant *P*-value of 0.1. In general, it is also possible to calculate a 95% confidence interval of the difference in the response rates for the high and the low doses (Gardner & Altman, 1986; Altman, 1991), but for the results of Cortesina *et al.* (1994) the most commonly used formula is inaccurate because of the small response rates and small sample sizes, and a more elaborate method would be required (Peskun, 1993).

So although Cortesina *et al.* found in their samples that regression occurred with a low but not with a high dose of interleukin 2, they cannot conclude that this will be true in general. Instead, a more accurate and informative summary statement is that 'four responses occurred in the 16 patients given the low dose (response rate 25%, 95% CI 7–53%) and no responses in the 15 patients given the higher dose (95% CI for response of 0–22%), difference not significant, $P = 0.1$'.

yours etc,

I.R. Campbell

### References

ALTMAN, D.G. (1991). *Practical Statistics for Medical Research*. Chapman & Hall: London.

ALTMAN, D.G. & MACHIN, D. (1993). Current statistical issues in clinical cancer research. *Br. J. Cancer*, **68**, 455–456.

CAMPBELL, I.R. (1992). *Statistical Analysis of End-Points in Cancer Clinical Trials*. MD Dissertation: Cambridge.

CORTESINA, G., DE STAFANI, A., GALEAZZI, E., CAVALLO, G.P., BADELLINO, F., MARGARINO, G., JEMMA, C. & FORNI, G. (1994). Temporary regression of recurrent squamous cell carcinoma of the head and neck is achieved with a low but not with a high dose of recombinant interleukin 2 injected perilymphatically. *Br. J. Cancer*, **69**, 572–576.

GARDNER, M.J. & ALTMAN, D.G. (1986). Confidence intervals rather than P values: estimation rather than hypothesis testing. *Br. Med. J.*, **292**, 746–750.

NEAVE, H.R. (1981). *Elementary Statistical Tables*. Unwin Hyman: London.

PESKUN, P.H. (1993). A new confidence interval method based on the normal approximation for the difference of two binomial probabilities. *J. Am. Stat. Assoc.*, **88**, 656–661.